# Primary B-Cell Deficiencies Reveal a Link between Human IL-17-Producing CD4 T-Cell Homeostasis and B-Cell Differentiation

**DOI:** 10.1371/journal.pone.0022848

**Published:** 2011-08-03

**Authors:** Rita R. Barbosa, Sara P. Silva, Susana L. Silva, Alcinda Campos Melo, Elisa Pedro, Manuel P. Barbosa, M. Conceição Pereira-Santos, Rui M. M. Victorino, Ana E. Sousa

**Affiliations:** 1 Instituto de Medicina Molecular, Faculdade de Medicina, Universidade de Lisboa, Lisboa, Portugal; 2 Hospital de Santa Maria, Centro Hospitalar Lisboa Norte, Lisboa, Portugal; Centre de Recherche Public de la Santé (CRP-Santé), Luxembourg

## Abstract

IL-17 is a pro-inflammatory cytokine implicated in autoimmune and inflammatory conditions. The development/survival of IL-17-producing CD4 T cells (Th17) share critical cues with B-cell differentiation and the circulating follicular T helper subset was recently shown to be enriched in Th17 cells able to help B-cell differentiation. We investigated a putative link between Th17-cell homeostasis and B cells by studying the Th17-cell compartment in primary B-cell immunodeficiencies. Common Variable Immunodeficiency Disorders (CVID), defined by defects in B-cell differentiation into plasma and memory B cells, are frequently associated with autoimmune and inflammatory manifestations but we found no relationship between these and Th17-cell frequency. In fact, CVID patients showed a decrease in Th17-cell frequency in parallel with the expansion of activated non-differentiated B cells (CD21^low^CD38^low^). Moreover, Congenital Agammaglobulinemia patients, lacking B cells due to impaired early B-cell development, had a severe reduction of circulating Th17 cells. Finally, we found a direct correlation in healthy individuals between circulating Th17-cell frequency and both switched-memory B cells and serum BAFF levels, a crucial cytokine for B-cell survival. Overall, our data support a relationship between Th17-cell homeostasis and B-cell maturation, with implications for the understanding of the pathogenesis of inflammatory/autoimmune diseases and the physiology of B-cell depleting therapies.

## Introduction

CD4 T cells with the ability to produce the pro-inflammatory cytokine interleukin (IL)-17, designated Th17 [1], [2], [3], act as co-ordinators of the innate and adaptive immune responses to bacteria and fungi, in particular *Candida albicans* [4], and have been implicated in several autoimmune diseases, such as multiple sclerosis, rheumatoid arthritis, systemic lupus erythematosus, psoriasis and Crohn's disease [5]. Common Variable Immunodeficiency Disorders (CVID) are defined by impaired antibody production and frequently associate with autoimmune and inflammatory manifestations [6], [7], [8], [9]. It is thus plausible that IL-17 may play a role in these processes.

The defects in mature B-cell development that characterize CVID mainly result in impaired organization of germinal centres (GC) [10], specialized structures within follicles where antigen-driven somatic hypermutation and class switch recombination occur, and thus the main source of switched-memory B cells and plasma cells [11]. Several molecular cues that are essential for B-cell differentiation in GCs are also required or may contribute to the induction and/or survival of Th17 cells. IL-6, a major factor for the differentiation of naive CD4 T cells into Th17 cells [3], also plays a key role in B-cell proliferation and antibody secretion [12]. IL-21 was first described as having a critical role in the regulation of antibody production by B cells [13], [14], and was later shown to be involved in Th17-cell differentiation [15], [16]. Furthermore, IL-21 is abundantly produced by Th17 cells and plays an important autocrine role in their differentiation and maintenance [4]. Several co-stimulatory molecules have also been shown to play roles in both Th17 induction and/or survival as well as in B-cell differentiation into plasma and memory B cells, namely ICOS and CD40L [17], [18], [19], [20], [21], [22]. T-cell help is known to be fundamental to the induction and subsequent organization of GCs, enabling an adequate generation of plasma and memory B cells. This help is a characteristic of a particular subset of T cells, follicular helper T cells (T_FH_), identifiable by the expression of the chemokine receptor CXCR5, which is essential for their specific homing to follicles in lymphoid tissues, and by the production of IL-21 [23]. Although T_FH_ cells reside mainly within follicles and GCs, a population of circulating T_FH_ cells has been consistently observed in humans [24], [25]. This circulating T_FH_ subset has been recently demonstrated to be a counterpart for T_FH_ cells found in GCs [26], and to be enriched not only in Th2 but also in Th17 cells that are able to help B-cell differentiation [26].

We hypothesized that the homeostasis of the circulating Th17 compartment may be related to B-cell differentiation. Confirming such a relationship would have major clinical implications, given the increasing use of B-cell depleting therapies in many autoimmune and lymphoproliferative diseases. As a strategy to investigate the contribution of B cells to the Th17 subset, we studied this population in CVID patients as well as in patients lacking B cells due to Congenital Agammaglobulinemia. This latter condition is associated with impaired early B-cell development in the bone marrow as a result, in the majority of cases, of mutations in the Bruton's tyrosine kinase gene, usually leading to a complete lack of circulating B cells [27]. The evaluation of these primary B-cell deficiencies combined with the study of healthy individuals supports a link between the homeostasis of the circulating Th17-cell pool and B-cell differentiation.

## Materials and Methods

### Ethics Statement

All subjects gave written informed consent for blood sampling and processing. The study was approved by the Ethical Board of the Faculty of Medicine of Lisbon.

### Participants

The study involved 30 healthy individuals, 31 patients with CVID, and 6 patients with Congenital Agammaglobulinemia. The clinical-epidemiological data of the three groups are summarized in Table 1. CVID patients were diagnosed according to the European Society for Immunodeficiency criteria (www.esid.org), namely decreased serum IgG as well as IgM and/or IgA levels at least 2 SD below the mean for age, impaired antibody response to vaccines, absent/low isohemagglutinins, and exclusion of defined causes of hypogammaglobulinemia. The 31 CVID patients were not related, with the exception of two homozygous twins. All the patients with Congenital Agammaglobulinemia had less than 1% B cells within total peripheral lymphocytes. 29 CVID and all Congenital Agammaglobulinemia patients were under IgG replacement therapy, adjusted to guarantee pre-infusion Ig levels above 650 mg/dL. The two CVID patients not receiving IgG had levels of total serum IgG of 227 and 473 mg/dL.

**Table 1:** Clinical and epidemiological data of the cohorts studied.

	Healthy	CVID	CongenitalAgammaglobulinemia^a^
Number (male/female)	30 (9/21)^d^	31 (11/20)	6 (6/0)
Age (*yrs.*)	41±15^d^	40±13	24±5
Clinical manifestations^b^^,^ c			
*Autoimmune disease*	n.a.	17/31 (55%)	0
*Adenopathies*	n.a.	10/31 (32%)	0
*Lymphoid proliferation*	n.a.	18/20^e^ (86%)	0
*Granulomas*	n.a.	3/20^e^ (15%)	0
*Chronic diarrhoea*	n.a.	15/31 (48%)	0
*Splenomegaly*	n.a.	16/31 (52%)	0
IgG replacement therapy^c^			
*intravenous*	n.a.	24/31 (77%)	6 (100%)
*subcutaneous*	n.a.	5/31 (16%)	0
Length of IgG therapy (*yrs.*)	n.a.	7±6	15±9

n.a. not applicable, CVID: Common Variable Immunodeficiency Disorders.

a Genetic defects in the *Btk* gene were identified in 4 of the congenital agammaglobulinemia patients, namely *IVS17-1G→C*, R288Q, *IVS8-2A→G*, and Y375X mutations; in the other 2 patients, *Btk* mutations have been excluded and other genes are currently being evaluated.

b Diagnostic criteria: Autoimmune disease - clinical data, given the impairment in Ab production; Adenopathies - lymph node larger than 1 cm diameter in 2 or more lymphatic chains in clinical and/or imaging exams; Lymphoid proliferation and Granulomas - diffuse lymphocytic infiltrates or granulomas on gastrointestinal, lymph node or pulmonary biopsies; Splenomegaly - longitudinal spleen diameter superior to 15 cm (computed tomography or ultrasonography).

c Percentage within total cohort evaluated in brackets.

d 15/30 healthy subjects were included in detailed immunological studies (10 female; age 39±11 years).

e Total number of individuals with biopsies.

### Monoclonal antibodies used in flow cytometry

The following anti-human monoclonal antibodies were used, with clone and the directly conjugated fluorochrome specified in brackets: CD3 (SK7; peridinin chlorophyll protein (PerCP)), CD4 (SK3; PerCP), CD8 (SK1; PerCP and allophycocyanin (APC)-Cy7; RPA-T8; APC), CCR6 (11A9; phycoerythrin (PE)), CD38 (HB7; PE), CD45RA (L48; PE-Cy7), IgD (IA6-2; PE), IgM (G20-127; APC), HLA-DR (L243; fluorescein isothiocyanate (FITC)), IFN-γ (4S.B3; FITC), IL-2 (MQ1-17H12; PE), IL-4 (MP4-25D2; PE), from BD Biosciences, San Jose, CA; CD3 (UCHT1; APC-eFluor780), CD4 (RPA-T4, FITC, PerCP-Cy5.5 and PE-Cy7), CD8 (RPA-T8; FITC and PE), CD19 (HIB19; PerCP-Cy5.5 and PE-Cy7), CD27 (O323; FITC, PE, APC and PE-Cy7), CD45RO (UCHL1; PE), CD69 (FN50; FITC), CD45RA (HI100; FITC and APC), IL-17A (eBio64DEC17; PerCP-Cy5.5 and Alexa Fluor 647), TNF-α (MAb11; PE), from eBiosciences, San Diego, CA; CD4 (S3.5; PE) from Caltag, Buckingham, UK; CCR7 (150503; FITC), CXCR5 (51505.111; PE), from R&D Systems, Minneapolis, MN; CD21 (BL13; FITC) from IO Test, Beckman Coulter, Brea, CA.

### Cell staining and flow cytometric analysis

Phenotypic analysis was performed in whole blood samples collected immediately before IgG administration, after staining with monoclonal antibodies and red blood cells lysis using BD FACS Lysing Solution (BD Biosciences). Samples were acquired on a FACSCalibur or on a FACSCanto flow cytometer (BD Biosciences). A minimum of 100,000 lymphocytes were acquired per sample. Data were analysed using CellQuest Software (BD Biosciences) and FlowJo Software (Tree Star Inc., Ashland, OR). Total cell numbers were calculated by multiplying the percentage of each population within total lymphocytes by the peripheral blood lymphocyte count obtained at the clinical laboratory on the day of sampling.

### Analysis of cytokine production

Freshly isolated peripheral blood mononuclear cells (PBMC) by Ficoll-Hypaque density gradient (Amersham Pharmacia Biotech, Uppsala, Sweden) were assessed for cytokine production at the single-cell level, as previously described [28]. Briefly, after a 4-hour culture with phorbol myristate acetate (PMA) (50 ng/mL, Sigma-Aldrich) plus ionomycin (500 ng/mL; Calbiochem, Merck Biosciences, Nottingham, U.K.), in the presence of brefeldin A (10 µg/mL; Sigma-Aldrich), PBMC were surface stained and then fixed (2% formaldehyde; Sigma-Aldrich, St Louis, MO), permeabilized (phosphate buffered saline/1% bovine serum albumin/0.5% saponin) (Sigma-Aldrich) and stained intracellularly with monoclonal antibodies against IL-2, IL-4, IFN-γ, TNF-α and IL-17. Flow cytometric analysis was subsequently performed as described above.

### Quantification of serum levels of BAFF

Serum concentrations of BAFF (BlyS/TNFSF13B; B-cell activating factor) were quantified by Enzyme Linked Immunosorbent Assay (ELISA) using the BAFF Immunoassay Kit (R&D Systems), according to the manufacturer's instructions.

### Statistical analysis

Statistical analyses were performed using GraphPad Prism version 5.0 (GraphPad Software Inc, SD). Two group comparisons were performed using Mann-Whitney test. Spearman's coefficient was used to determine the significance of the correlation between two variables. Results are expressed as mean±SEM, and *P*-values<0.05 were considered to be significant.

## Results

### Th17 cells in patients with CVID

**Figure 1:**
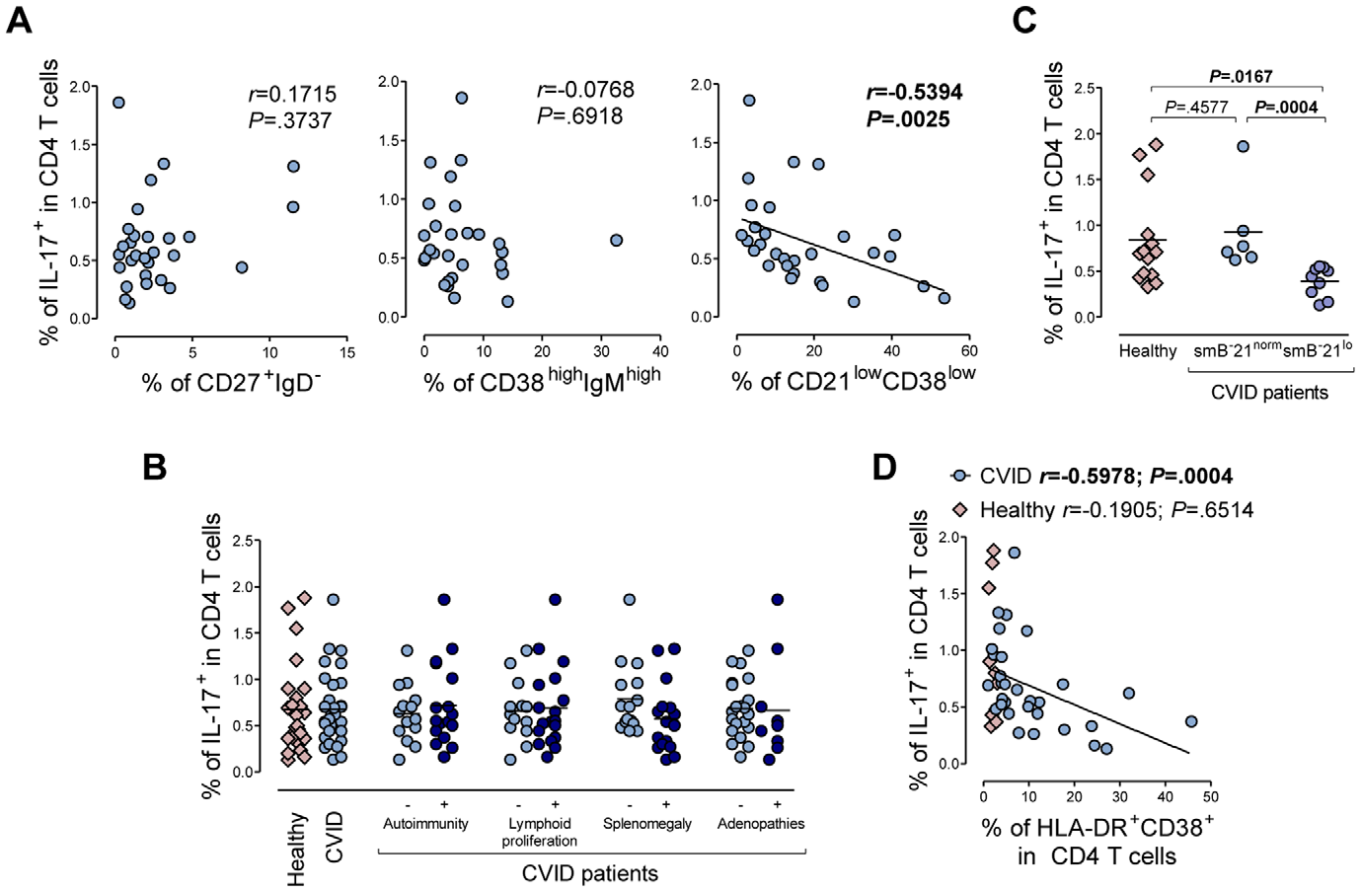
Th17 cells in patients with CVID. (A) Correlation between Th17 frequency and frequency of switched-memory B cells (left), transitional B cells (middle) or CD21^low^ B cells (right), in CVID patients. Switched-memory B cells were defined as CD27^+^ IgD^−^ cells, transitional B cells as CD38^high^ IgM^high^ cells and CD21^low^ B cells were defined as CD21^low^ CD38^low^ cells within gated B cells (CD19^+^) after surface staining of whole blood samples. IL-17 expression was assessed at the single-cell level by intracellular staining following short-term stimulation of PBMC with PMA and ionomycin. (B) Th17 frequency in CVID individuals stratified according to their clinical manifestations, namely autoimmunity, lymphoid proliferation, splenomegaly, and adenopathies. (C) Th17 frequency in healthy controls and CVID patients grouped according to EUROclass. (D) Correlations between frequencies of activated CD4 T cells, defined by concurrent expression of HLA-DR and CD38, and of Th17 cells in CVID and healthy individuals. Each symbol represents one individual. Bars represent mean. Data were compared using Mann-Whitney test, and *P* values are shown. Correlation significance was assessed using Spearman coefficient test, and *r* and *P* values are shown.

We evaluated, for the first time, the frequency of circulating Th17 cells in CVID. Our CVID cohort (Table 1) featured the characteristic impairment of GC organization and generation of B-cell memory, demonstrated by the striking decrease in the frequency of switched-memory B cells, accumulation of CD38^hi^IgM^hi^ transitional B cells, and expansion of CD21^low^CD38^low^ B cells, a population believed to be related to continuous B-cell activation in the presence of impaired GC function [29], [30] (Figure S1A and S1B). No association was observed between the frequency of Th17 cells and transitional B cells, a pre-GC B-cell population (Figure 1A). In contrast, we found a statistically significant negative correlation between Th17 cells and CD21^low^CD38^low^ B cells, in CVID patients (Figure 1A), suggesting a decline in the frequency of circulating Th17 cells that matched the B-cell disturbances indicative of GC disruption. The fact that we did not observe a relationship with the frequency of switched-memory B cells is likely related to the heterogeneity of blocks in GC differentiation that CVID patients present [10]. Our CVID cohort presented an unusually high proportion of autoimmune and lymphoproliferative manifestations (Table 1), possibly related to a reference bias associated with an immunology department in a central hospital. We next assessed whether these inflammatory and autoimmune manifestations could be related to an expansion of Th17 cells, as reported in other autoimmune settings [5]. We found no increase in the frequency of Th17 cells in CVID patients with autoimmunity (Figure 1B), even when CVID patients were split according to the type of autoimmune manifestation, namely autoimmune cytopenias and organ specific autoimmunity (data not shown). In contrast, the CD21^low^CD38^low^ B-cell subset was significantly expanded in the subgroup of CVID patients with autoimmunity (Figure S1C).

CVID patients have been classified according to the previously mentioned B-cell sub-populations [9]. The stratification of our CVID cohort according to the EUROclass classification [9] revealed that a statistically significant decrease in Th17 frequency was restricted to the group with less than 2% switched-memory B cells and more than 10% CD21^low^CD38^low^ B cells (Figure 1C), in agreement with the above results.

Several T-cell imbalances were also observed in the CVID cohort, as previously reported [31], namely an expansion of effector-memory T cells, increased production of the pro-inflammatory cytokines interferon (IFN)-γ and tumour necrosis factor (TNF)-α, and up-regulation of activation markers within both CD4 and CD8 T cells (Figure S2). Of note, the frequency of Th17 cells was found to negatively correlate with CD4 T-cell activation in CVID patients (Figure 1D), with no relationship with naïve/memory T-cell imbalances (correlation with the frequency of naïve cells within CD4 T cells, *r* = 0.3065, *P* = .1058). In contrast, the frequency of IFN-γ-producing cells was found to negatively correlate with naïve CD4 T cells (*r* = −0.8199, *P*<.0001) and positively with the levels of CD4 T-cell activation in CVID patients (*r* = 0.6098, *P* = .0003). Similar relationships were observed with the expression levels of activation markers within the CD8 T-cell subset (data not shown). We also investigated whether the increased production of IFN-γ observed in CVID patients had an impact on the concomitant production of this cytokine by Th17 cells. We found no significant differences in the proportion of IFN-γ^+^ cells within the IL-17-producing CD4 subset as compared to healthy subjects (22.55%±2.34% in CVID and 14.45%±1.70% in healthy, *P* = .0567) and no relationship between the frequencies of IFN-γ- and IL-17-producing CD4 T cells (r = −0.2504, *P* = .1821). Moreover, in contrast to IFN-γ-producing CD4 T cells, no association was found between the frequency of IL-17^+^IFN-γ^+^ CD4 T cells and the naïve/memory imbalances (*P*>.1500).

In conclusion, no association between Th17 cells and autoimmune manifestations or production of other pro-inflammatory cytokines was observed in CVID patients. The levels of Th17 cells were apparently more directly related to B-cell differentiation than to T-cell disturbances, showing a negative correlation with the pathological expansion of a B-cell population associated with impaired GC function (CD21^low^CD38^low^ B cells) in CVID.

### Decreased frequency of Th17 cells in individuals without B cells

**Figure 2:**
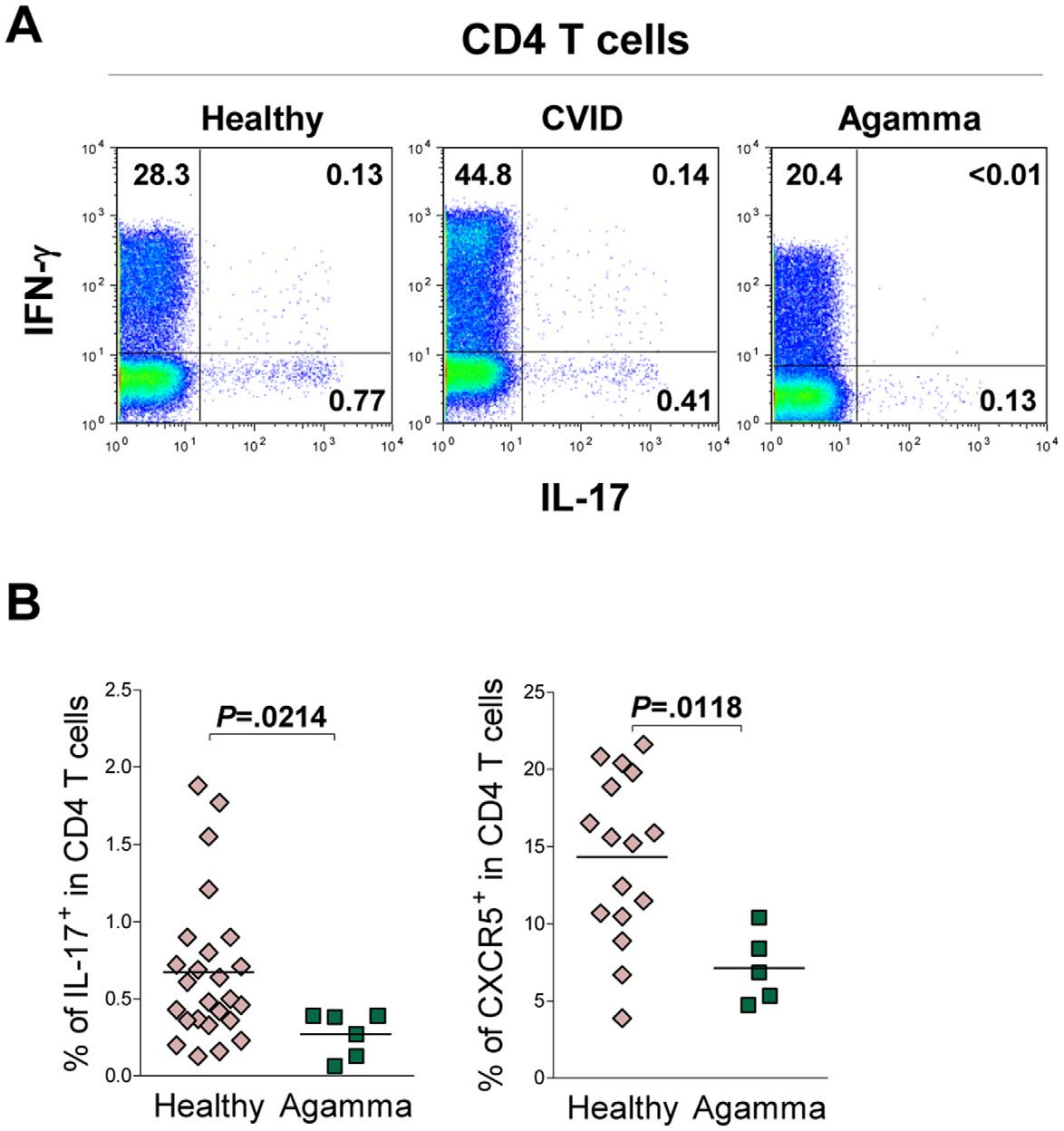
Decreased frequency of Th17 cells in individuals lacking B cells. (**A**) Representative dot-plots of the analysis of IFN-γ and IL-17 production by CD4 T cells, determined by intracellular staining, for a healthy individual (left), a CVID patient (middle) and a Congenital Agammaglobulinemia (Agamma) patient (right). Numbers inside dot-plots represent the proportion of cells expressing the markers. (B) Frequency of Th17 cells (left) and CXCR5^+^ CD4 T cells (right) in healthy individuals and Congenital Agammaglobulinemia patients (Agamma). Each symbol represents one individual. Bars represent mean. Data were compared using Mann-Whitney test, and *P* values are shown.

In order to further investigate the relationship between B cells and the IL-17-producing CD4 subset, we evaluated this population in individuals with Congenital Agammaglobulinemia, who lack mature B cells due to genetic defects impairing early B-cell development [27] (Table 1). A marked reduction in the frequency of Th17 cells was found in these patients (Figure 2A and 2B).

In spite of the reduced Th17 frequency, these patients do not exhibit a major increase in the frequency of infections with *Candida albicans*, which may be related to the preservation of innate sources of IL-17, such as γδ T cells, natural killer T cells or myeloid cells.

Of note, these individuals featured neither significant naïve T-cell imbalances, nor major alterations in the levels of T-cell activation nor frequencies of IFN-γ-, TNF-α-, IL-2- or IL-4-producing cells, as compared to healthy subjects (Figure S2).

Importantly, a significant reduction of circulating T_FH_ cells was found in these patients (Figure 2B), likely related to decreased GC generation in the context of Congenital Agammaglobulinemia, as recently reported [32]. A decrease in this circulating CD4 T-cell population has also been described in the context of human ICOS-deficiency, a situation in which the generation of GCs is known to be severely perturbed [33].

In conclusion, we report here a major reduction in the Th17-cell pool in patients lacking B cells.

### Direct correlation between the frequency of circulating Th17 cells and switched-memory B cells in healthy individuals

**Figure 3:**
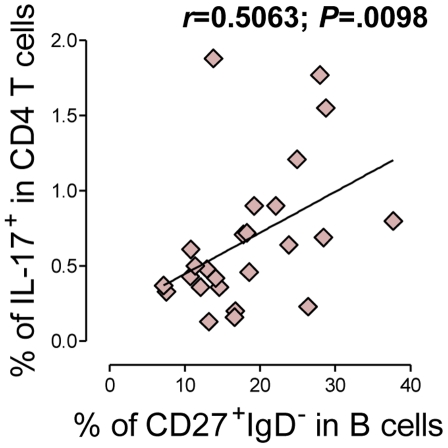
Direct correlation between the frequencies of circulating Th17 cells and switched-memory B cells in healthy individuals. Correlation between the frequencies of switched-memory (CD27^+^IgD^−^) B cells and Th17 cells in healthy individuals. Each symbol represents one healthy individual. Correlation significance was assessed using Spearman coefficient test, and *r* and *P* values are shown.

Our findings suggesting a relationship between the homeostasis of the circulating Th17 compartment and B-cell disturbances led us to investigate this putative relationship in healthy individuals. We found a direct correlation between the frequency of CD4 T cells able to produce IL-17 and the frequency of B cells exhibiting a switched-memory phenotype in these individuals (Figure 3). This relationship was also observed when absolute numbers of the circulating populations were considered (r = 0.4565, *P* = .0249).

**Figure 4:**
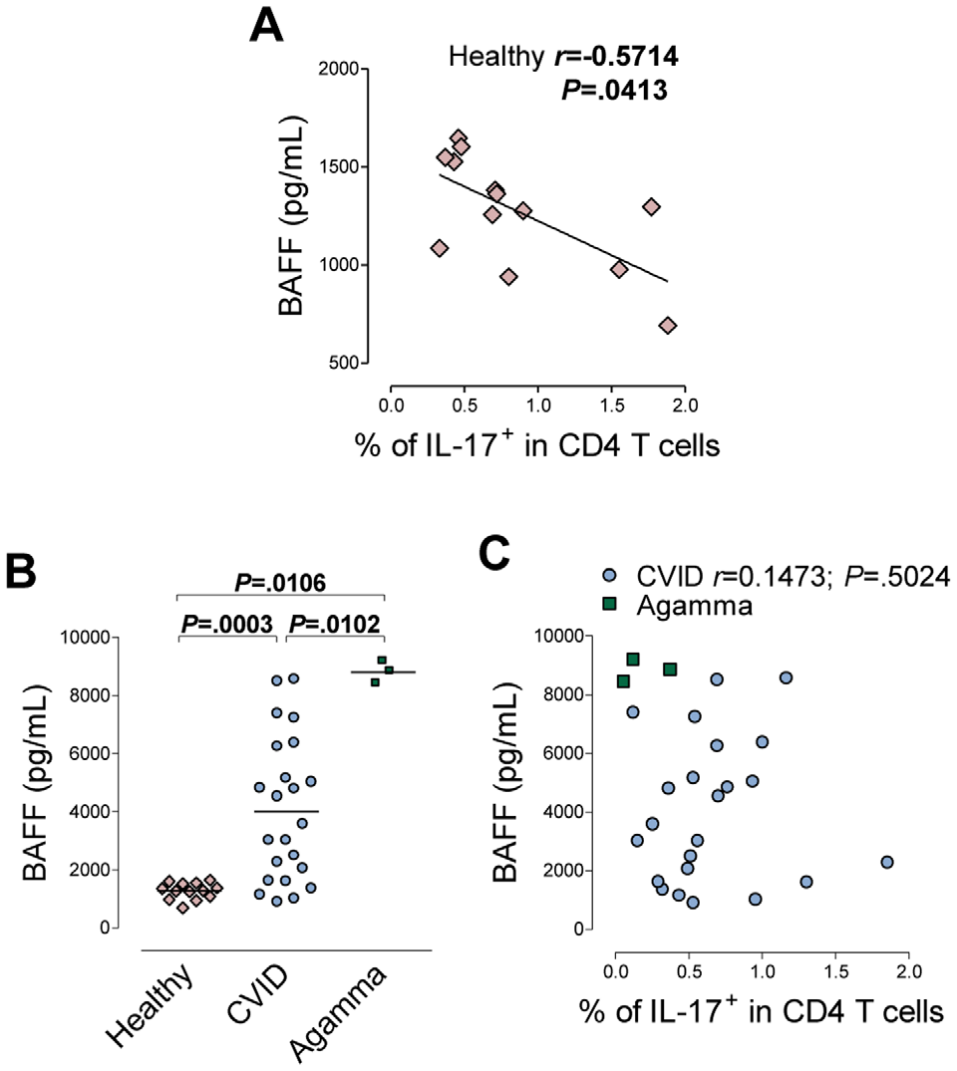
Negative correlation between the frequency of Th17 cells and serum BAFF levels in healthy subjects. (A) Correlation between the frequency of Th17 cells and serum levels of the cytokine BAFF, as determined by ELISA, in healthy individuals. (B) Analyses of the serum levels of BAFF in healthy individuals, CVID and Congenital Agammaglobulinemia patients. Each symbol represents one individual. Data were compared using Mann-Whitney test, and *P* values are shown. (C) The same correlation described in (A) for CVID patients and Congenital Agammaglobulinemia patients. Each symbol represents one individual. Correlation significance was determined using Spearman coefficient test, and *r* and *P* values are shown.

In addition, healthy individuals exhibited a negative correlation between the frequency of Th17 cells and the serum levels of B-cell activating factor (BAFF) (Figure 4A), a critical cytokine for B-cell differentiation and survival [34]. In contrast, we did not observe any relationship between BAFF serum levels and the production of other pro-inflammatory cytokines, such as IFN-γ and TNF-α, or IL-4 (data not shown), thus highlighting the specificity of the link to Th17 cells. These results are in conformity with our previous observation of a relationship between IL-17 production by CD4 T cells and B-cell maturation in the context of primary B-cell deficiencies.

It has recently been demonstrated in mice that local BAFF-gene targeting in dendritic cells suppressed both the generation of plasma cells and Th17 cells [35], suggesting that BAFF promotes Th17-cell proliferation and expansion, possibly through the modulation of the cytokine milieu. However, providing our finding of a negative association between BAFF and IL-17 we hypothesize that BAFF effects may be indirectly mediated by the B-cell subset. In order to further investigate this possibility we quantified serum BAFF levels in the CVID and Congenital Agammaglobulinemia cohorts. In agreement with recent reports [36], [37], serum BAFF levels were significantly increased in CVID patients, and were even higher in Congenital Agammaglobulinemia, reaching statistical significance in comparison to both healthy individuals and to CVID patients (Figure 4B). However, no correlation was found between BAFF serum levels and the frequency of Th17 cells in CVID and Congenital Agammaglobulinemia patients (Figure 4C). Notably, in the absence of B cells as illustrated by Congenital Agammaglobulinemia patients, very high levels of BAFF may be associated with very low frequencies of Th17 cells (Figure 4C), not supporting a direct role of BAFF in the induction/survival of Th17 cells. Overall, our data suggest that the association between IL-17-producing CD4 T cells and BAFF is likely related to its effects on B cells.

## Discussion

We report here an association between the frequencies of Th17 cells and switched-memory B cells in healthy individuals, and a marked reduction of this CD4 subset in patients with congenital absence of peripheral B cells. Additionally, in patients with primary defects in mature B-cell differentiation, the frequency of Th17 cells was inversely correlated with markers of GC impairment, further supporting the link between B cells and the differentiation or maintenance of Th17 cells.

Our data generated in human B-cell deficiencies suggest, for the first time, that B cells play a role in the homeostasis of the Th17 subset. On the other hand, several previous reports have implicated IL-17 in B-cell differentiation and function. A recent report [38] suggested that IL-17 and possibly Th17 cells can contribute to GC function in general. This work using BXD2 mice, which develop spontaneous erosive arthritis associated with auto-antibody production, showed that the enhanced somatic hypermutation and class-switch recombination found in these animals resulted from the direct action of IL-17 on B cells, leading to increased frequency and duration of GCs [38]. Recent data from human studies also suggest that Th17 cells may contribute to the generation of ectopic GCs within kidney allografts through the production of IL-21 [39], further establishing a link between IL-17 and B-cell function. Importantly, IL-17 alone has been described to promote human B-cell survival and to synergize with BAFF to induce B-cell proliferation and differentiation into antibody-secreting plasma cells [40]. Recent reports have, in this sense, established a direct impact of IL-17 on B cells [38], [40], with respect to proliferation, survival and antibody production, and have shown that Th17 cells can act as B-cell helpers [41]. Our observation of markedly reduced levels of Th17 cells in the absence of other major T-cell imbalances in patients with congenital absence of B cells suggests an important contribution of B cells to the homeostasis of Th17 cells. Although the number of patients with this rare immunodeficiency under follow-up is small, the remarkable consistency of the results generated in these patients strengthens our finding of a reduced Th17 compartment in Congenital Agammaglobulinemia. Such a relationship was further supported by the negative correlation between Th17 frequency and the expansion of the CD21^low^CD38^low^ B-cell subset in CVID patients, a pathological B-cell population resulting from altered B-cell activation associated with defects in GC function [29], [30].

Our results raise the possibility of a follicular contribution to the maintenance of the Th17-cell subset, a notion further supported by a recent report [26]. We were able to reveal a fraction of Th17 cells expressing both CXCR5 and CCR7, in addition to CCR6, likely having the potential to home to follicles (data not shown). This is particularly relevant given the fact that circulating T_FH_ cells were reported to be enriched in Th17 cells that are able to help B-cell differentiation [26]. We have studied this population in patients without peripheral B cells due to early defects in B-cell development, which, as described in ICOS-deficient patients [33], [42], are expected to have reduced generation of GCs. We found the circulating CXCR5^+^ CD4 T-cell population to be significantly diminished in these patients, as compared to healthy subjects, in agreement with a recent report [32], further supporting the notion of circulating T_FH_ cells being counterparts of the follicular helper T cells found in GCs. Our results suggest that the impairment in GC generation may underlie the reduced frequencies of both T_FH_ and Th17 cells.

Contrary to what we observed in Congenital Agammaglobulinemia, CVID patients presented with several T-cell imbalances [31], namely an increase in the production of the pro-inflammatory cytokines IFN-γ and TNF-α, in direct correlation with the up-regulation of activation markers. We found that the Th17-cell population decreased concurrently with the hyper-activated state, suggesting that a different mechanism is involved in its regulation in CVID patients. Furthermore, a significant proportion of our CVID cohort featured autoimmune manifestations and some patients exhibited inflammatory processes that shared similarities with the pathology of Crohn's disease, a condition in which Th17 cells have been implicated [5], [43]. However, in contrast to Crohn's disease, it has been shown that CVID patients with symptomatic gut inflammation exhibit a reduced ability to produce IL-17 and IL-23 by the lamina propria mononuclear cells, in spite of increased IL-12 and IFN-γ secretion [43]. Importantly, we found no increase in the frequency of CD4 T cells able to produce IL-17 in CVID patients presenting with autoimmune disease, either with autoimmune cytopenia or other autoimmune disorders. In fact, an inverse correlation was found with the expansion of the CD21^low^CD38^low^ B-cell subset that has been implicated in autoimmunity. These findings are particularly relevant given that our cohort presents an exceptionally high prevalence of autoimmune manifestations.

A recent report suggested that the commercial immunoglobulin (Ig) used in intravenous therapy has the ability to inhibit the differentiation, amplification and function of human Th17 cells *in vitro* [44]. However, such an effect is unlikely to account for the observations described here, since both Congenital Agammaglobulinemia and CVID patients undergo replacement therapy with Ig (Table 1).

The development and homeostasis of Th17 cells and memory B cells share several determinants. Tumour growth factor (TGF)-β has a unique role in driving IgA isotype switching, which is the major Ig type produced at mucosal sites [45], and is also critical for Th17-cell differentiation [15], [16]. Both IgA and IL-17-producing CD4 T cells have an important function in the modulation of gut flora and mucosal immunity [45]. It is thus plausible that the link between IL-17 production and B-cell function could mainly lie in the isotype switch to IgA, a notion further supported by the fact that patients with Congenital Agammaglobulinemia or CVID have impaired IgA production. This possibility led us to investigate the frequency of circulating Th17 cells in patients with selective IgA deficiency. However, no imbalances in this CD4 subset were found in patients with undetectable serum levels of IgA as compared to healthy individuals (data not shown), suggesting that the link between B cells and IL-17 production is not dependent of the development of IgA-producing cells. It is unlikely that a unique molecule or pathway determine the impact of B cells in the homeostasis of the Th17 subset, being more plausible that several mechanisms are involved, either through direct interactions or through the influence of factors modulated by B cells.

BAFF is a crucial survival factor for peripheral B cells. As expected from previous reports, both CVID and Congenital Agammaglobulinemia patients presented highly increased serum levels of this cytokine [36], [37]. Although increased BAFF production by cells of the innate immune system, such as neutrophils, macrophages, monocytes and dendritic cells, could be enhanced in these patients, contributing to these increased levels, it is more likely that they resulted from reduced consumption of BAFF due to the decreased number of target cells in both conditions. This latter hypothesis is supported by the fact that B-cell depletion upon rituximab treatment, a monoclonal antibody targeting CD20, results in significantly increased levels of BAFF that return to baseline levels upon B-cell re-population [46]. Thus, high BAFF levels may be considered a measure of B-cell dysfunction and, in this sense, the negative correlation we report in healthy individuals between the frequency of Th17 cells and the serum levels of BAFF further strengthens the link between IL-17 production and B-cell maturation.

B cell-depleting therapies have been increasingly used in various clinical settings, including pathologies where IL-17 has been implicated [46], [47], [48]. Additionally, BAFF-targeting therapies offer a promising approach for autoimmune/inflammatory disorders [46], [48]. Our data raise the possibility that these treatments may have an impact on Th17 cells and, in this way, influence the clinical outcome mediated by these therapeutic strategies. In line with these observations, a very recent report has shown that rituximab treatment selectively reduces the Th17-cell response in rheumatoid arthritis patients [49]. Nevertheless, such an inhibition does not seem to lead to an increased frequency of mucocutaneous candidiasis in rituximab-treated patients [49], an infection associated with Th17 deficiency.

In conclusion, our data show, for the first time, that the induction/proliferation/survival of Th17 cells is related to B-cell function. These results provide support for a link between Th17 and B cells that is relevant for the understanding of the pathogenesis of inflammatory/autoimmune diseases as well as the mechanisms underlying the effects of therapeutic strategies targeting B cells.

## Supporting Information

Figure S1 **B-cell disturbances in CVID patients.** (A) Representative plots of the flow cytometry analysis of switched-memory B cells (top), CD21^low^CD38^low^ B cells (middle) and transitional B cells (bottom), in healthy individuals (left panels) and CVID patients (right panels). Numbers represent the percentage of the given population within CD19^+^ cells. (B) Comparison of the frequencies of these B-cell subsets in CVID and in healthy individuals. (C) Frequency of CD21^low^CD38^low^ within B cells in healthy controls and CVID individuals stratified according to their clinical manifestations, namely autoimmunity, lymphoid proliferation, splenomegaly, and adenopathies. Each symbol represents one individual. Bars represent mean. Data were compared using Mann-Whitney test, and *P* values are shown.
(TIF)

Figure S2 **T-cell disturbances in CVID and Congenital Agammaglobulinemia patients.** Analysis of: (A) frequency and absolute numbers of naïve (CD45RA^+^CD27^+^) within CD4 T cells; (B) frequency and absolute numbers of naïve (CD45RA^+^CD27^+^) and terminally-differentiated (CD45RA^+^CD27^−^) within CD8 T cells; (C) frequency of activated (HLA-DR^+^CD38^+^) within CD4 T cells and CD8 T cells; and (D) frequencies of IFN-γ-, TNF-α-, IL-4-, and IL-2-producing CD4 T cells assessed at the single-cell level by intracellular staining following short-term PBMC stimulation with PMA and ionomycin. Bars represent mean±SEM. Data were compared using Mann-Whitney test, and *P* values are shown.
(TIF)
