# Supplementary figures and images for: Primary B-Cell Deficiencies Reveal a Link between Human IL-17-Producing CD4 T-Cell Homeostasis and B-Cell Differentiation
Source: PLoS One. 2011 Aug 3;6(8):e22848. doi: 10.1371/journal.pone.0022848 (PMC3149619; doi:10.1371/journal.pone.0022848)

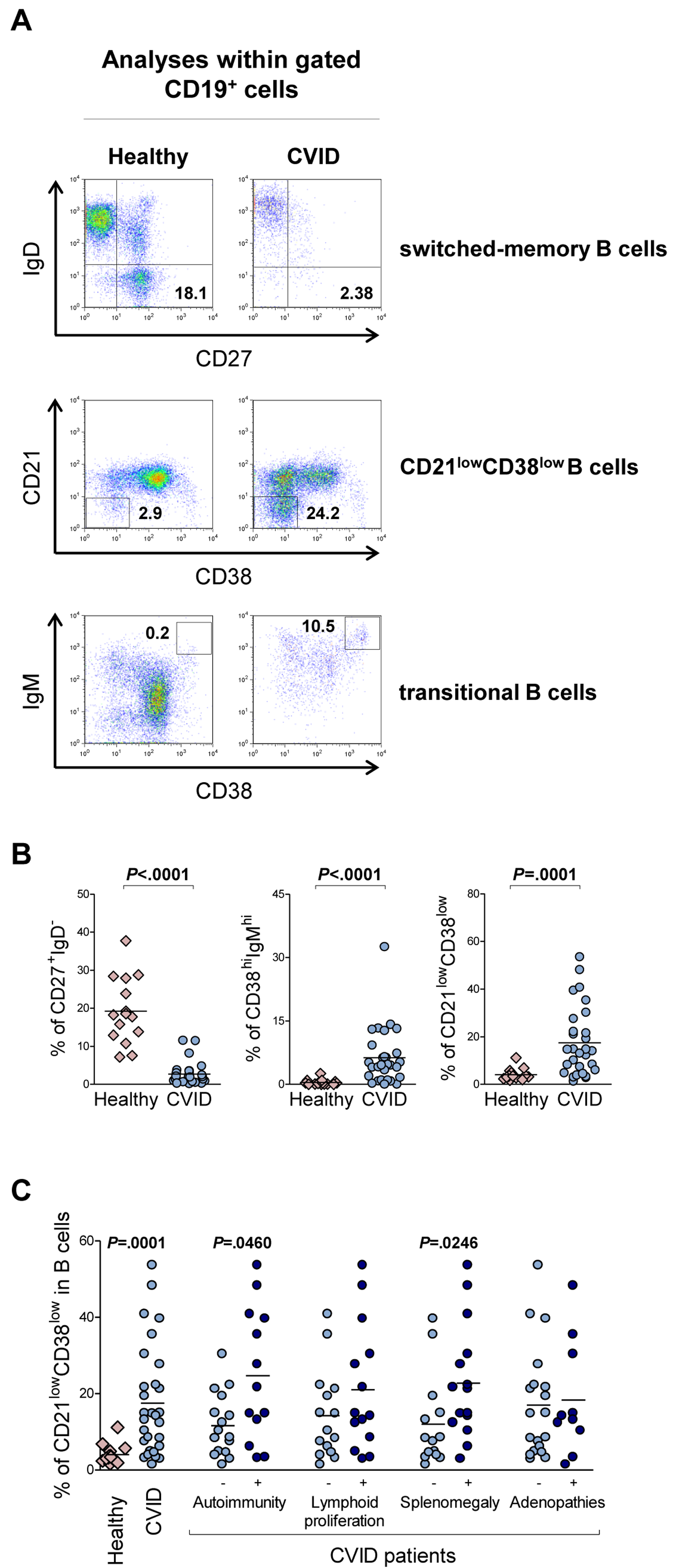

Supplement: Figure S1 — B-cell disturbances in CVID patients. (A) Representative plots of the flow cytometry analysis of switched-memory B cells (top), CD21lowCD38low B cells (middle) and transitional B cells (bottom), in healthy individuals (left panels) and CVID patients (right panels). Numbers represent the percentage of the given population within CD19+ cells. (B) Comparison of the frequencies of these B-cell subsets in CVID and in healthy individuals. (C) Frequency of CD21lowCD38low within B cells in healthy controls and CVID individuals stratified according to their clinical manifestations, namely autoimmunity, lymphoid proliferation, splenomegaly, and adenopathies. Each symbol represents one individual. Bars represent mean. Data were compared using Mann-Whitney test, and P values are shown. (TIF) [file pone.0022848.s001.tif]

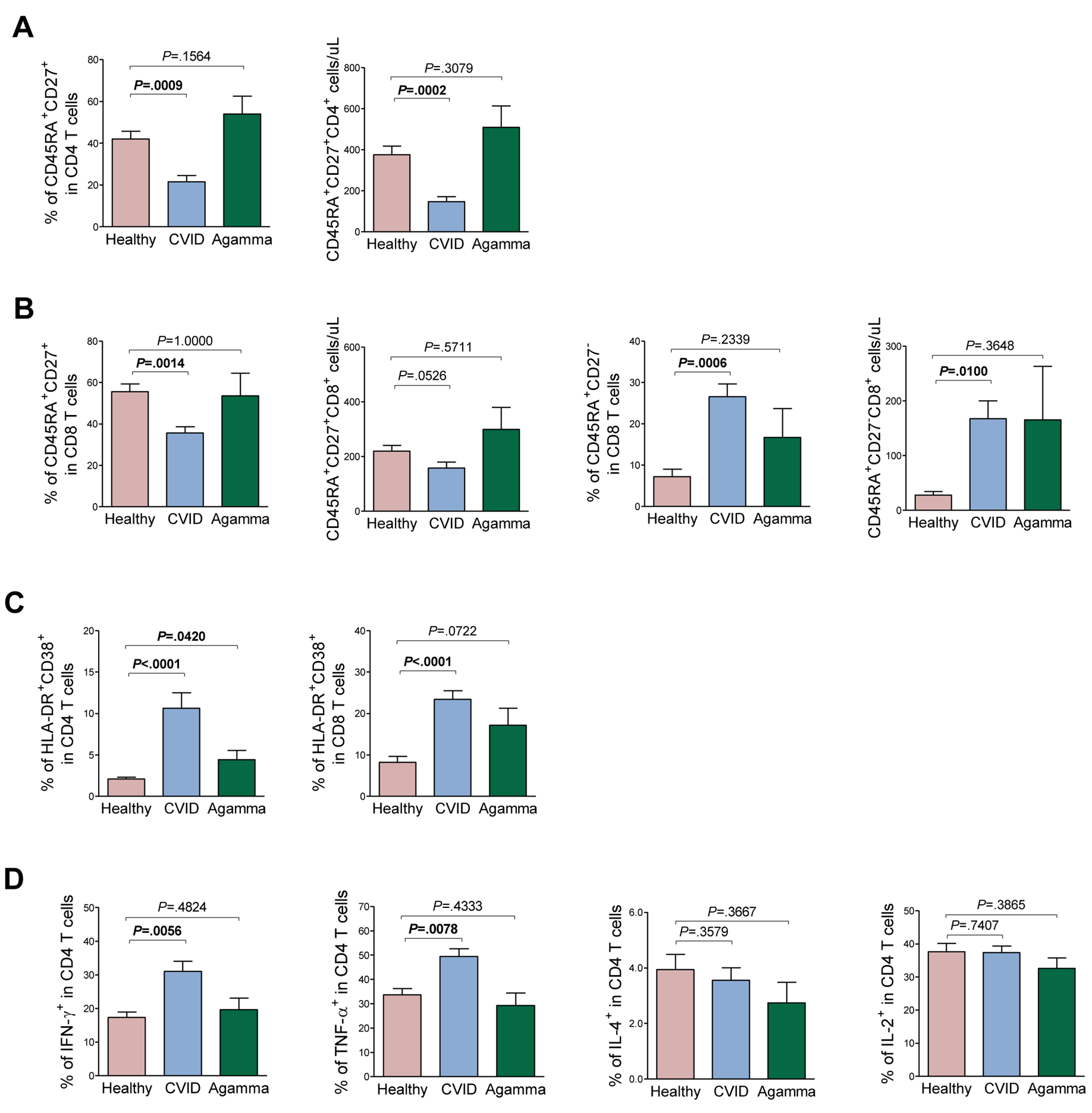

Supplement: Figure S2 — T-cell disturbances in CVID and Congenital Agammaglobulinemia patients. Analysis of: (A) frequency and absolute numbers of naïve (CD45RA+CD27+) within CD4 T cells; (B) frequency and absolute numbers of naïve (CD45RA+CD27+) and terminally-differentiated (CD45RA+CD27−) within CD8 T cells; (C) frequency of activated (HLA-DR+CD38+) within CD4 T cells and CD8 T cells; and (D) frequencies of IFN-γ-, TNF-α-, IL-4-, and IL-2-producing CD4 T cells assessed at the single-cell level by intracellular staining following short-term PBMC stimulation with PMA and ionomycin. Bars represent mean±SEM. Data were compared using Mann-Whitney test, and P values are shown. (TIF) [file pone.0022848.s002.tif]
